# Characterization and Functional Analysis of the Calmodulin-Binding Domain of Rac1 GTPase

**DOI:** 10.1371/journal.pone.0042975

**Published:** 2012-08-15

**Authors:** Bing Xu, Prashen Chelikani, Rajinder P. Bhullar

**Affiliations:** 1 Department of Oral Biology, University of Manitoba, Winnipeg, Manitoba, Canada; 2 Department of Biochemistry and Medical Genetics, University of Manitoba, Winnipeg, Manitoba, Canada; BioScience Project, United States of America

## Abstract

Rac1, a member of the Rho family of small GTPases, has been shown to promote formation of lamellipodia at the leading edge of motile cells and affect cell migration. We previously demonstrated that calmodulin can bind to a region in the C-terminal of Rac1 and that this interaction is important in the activation of platelet Rac1. Now, we have analyzed amino acid residue(s) in the Rac1-calmodulin binding domain that are essential for the interaction and assessed their functional contribution in Rac1 activation. The results demonstrated that region 151–164 in Rac1 is essential for calmodulin binding. Within the 151–164 region, positively-charged amino acids K153 and R163 were mutated to alanine to study impact on calmodulin binding. Mutant form of Rac1 (K153A) demonstrated significantly reduced binding to calmodulin while the double mutant K153A/R163A demonstrated complete lack of binding to calmodulin. Thrombin or EGF resulted in activation of Rac1 in CHRF-288-11 or HeLa cells respectively and W7 inhibited this activation. Immunoprecipitation studies demonstrated that higher amount of CaM was associated with Rac1 during EGF dependent activation. In cells expressing mutant forms of Rac1 (K153A or K153A/R163A), activation induced by EGF was significantly decreased in comparison to wild type or the R163A forms of Rac1. The lack of Rac1 activation in mutant forms was not due to an inability of GDP-GTP exchange or a change in subcelllular distribution. Moreover, Rac1 activation was decreased in cells where endogenous level of calmodulin was reduced using shRNA knockdown and increased in cells where calmodulin was overexpressed. Docking analysis and modeling demonstrated that K153 in Rac1 interacts with Q41 in calmodulin. These results suggest an important role for calmodulin in the activation of Rac1 and thus, in cytoskeleton reorganization and cell migration.

## Introduction

Small GTPases of the Rho family are important signaling proteins in eukaryotic organisms and regulate a variety of cellular processors such as reorganization of cytoskeleton, membrane trafficking and cell adhesion. Through these effects, Rho GTPases coordinate cell migration and neurite outgrowth, cancer invasion and metastasis [1], [2]. Rac1 is a member of the Rho family of small GTP-binding proteins and acts as a molecular switch, cycling between the inactive GDP-bound form and the active GTP-bound form. Guanine nucleotide factors (GEFs) and GTPase activating proteins (GAPs) are key regulators of small GTPases with GEFs promoting conversion to the active GTP bound state and GAPs promoting conversion to the inactive state by stimulating intrinsic rate of GTP hydrolysis to GDP [3].

In addition to its role in actin dynamics, Rac1 also promotes cell proliferation [4] and cell division [5]. Rac1 is up-regulated in human tumors including breast, lung and colon [6], and its activation can control plasticity of tumor cell movement [7]. Moreover, Rac1 is also required for nuclear translocation of STAT transcription factors and β-catenin [8]. Recently it has been reported that Rac1 can activate JNK1 and control nuclear localization of β-catenin in canonical Wnt signaling [9]. In human blood platelets, Rac1 is activated through GPCRs and phospholipase C activation and calcium was essential for this activation [10]. Genetic and pharmacologic evidence showed that Rac1 GTPase is involved in regulation of platelet secretion and aggregation [11]. Rac1 also plays a redundant and crucial role in T-cell development [12].

Ca^2+^ is an important intracellular secondary messenger in eukaryotes and mediates its effects to a variety of stimuli through the Ca^2+^ binding protein, Calmodulin (CaM) [13]. CaM has unique structure consisting of two globular domains possessing two helix-loop-helix Ca^2+^-binding motifs referred to as EF-hand that are linked by a central helix [14], [15]. The central helix allows CaM to interact with a variety of proteins. Binding of calcium to CaM leads to reorganization of the secondary structure of CaM and the helix becomes complete and flexible [16]. CaM can bind to at least 300 target proteins which are classified as Ca^2+^ dependent, Ca^2+^ independent, and Ca^2+^ inhibited proteins [16]. CaM as a cellular Ca^2+^ sensor regulates target proteins through three main mechanisms: relief of auto-inhibition, active site remodeling or dimerization of target domains [17], [18]. Recently, many studies have demonstrated that CaM binds and regulates the function and activity of several small GTPases [19]–[23].

We demonstrated previously that CaM can interact with the small GTPase Rac1 and regulate its activity in platelet [24]. However, the interaction between CaM and Rac1 has not been analyzed in detail. Further studies are still needed to characterize the binding domain and assess functional role of CaM binding domain in Rac1 activation. In this paper, we have analyzed the amino acid residues in the putative Rac1-CaM binding domain that are essential for interaction and analyzed the role of these amino acids in the CaM mediated activation of Rac1. The results demonstrated that amino acid K153 and R163 in the Rac1-CaM binding domain are necessary for interaction with CaM. Thrombin and EGF respectively resulted in the activation of Rac1 in CHRF-288-11 cells and HeLa cells respectively while W7 inhibited the activation of Rac1 by thrombin and EGF. The activation of K153A Rac1 mutant and K153A/R163A double mutant was significantly decreased compared to the wild type and R163A mutant. These results demonstrate a significant role for CaM in the function of Rac1.

## Materials and Methods

### Reagents and plasmids

Monoclonal Rac1 antibody was purchased from BD Transduction Laboratories. Monoclonal CaM antibody was from Upstate Biotechnologies. HA and GFP antibodies were purchased from Santa Cruz Biotechnology. Thrombin, glutathione–agarose beads and anti-HA antibody coupled to agarose beads were purchased from Sigma. Bovine brain CaM and W7•HCl were purchased from Calbiochem. Triton X-100, bovine serum albumin standard and Bio-Rad protein assay dye reagent were from Bio-Rad Laboratories. All other reagents were from Sigma except where indicated. ShRNA plasmids for CaMs were obtained from Human shRNA libraries (Open Biosystem). GST-Rac1, GST-PAK1 bacterial expression plasmids and pCMV-HA expression vector were kindly provided by Dr. R. Weinberg, Dr. M. Hoshino and Dr X. J. Yao respectively. The pcDNA3-EGFP-Rac1WT, pcDNA3-EGFP-Rac1 (Q61L) and pcDNA3-EGFP-Rac1 (T17N) were purchased from Addgene.

### Plasmid Construction and Mutagenesis

The versions of Rac1 with changes in the CaM binding region and CaM mutant Q41 were generated using site-directed mutagenesis kit (Stratagene, La Jolla, CA) and were as follows: truncated Rac1 (Δ151–164), Rac1 (K153A), Rac1 (R163A), Rac1 (K153A and R163A). The full-length GST-Rac1 was used as the template. The primer used to generate Rac1Δ151–164 were CATGGCTAAGGAGATTGGTCTCAAGACAGTGTTTGACG (forward) and CGTCAAACACTGTCTTGAGACCAATCTCCTTAGCCATG (reverse). The primer used to mutate Rac1(K153A) were GCCGAGCACTCCAGGTATGCTACAGCA (forward) andAAGGAGATTGGTGCTGTAGCATACCTG (reverse). The primer used to mutate (R163A) were CGGCGCTCACACAGGCAGGCCTCAAGACAG (forward) and CTGTCTTGAGGCCTGCCTGTGTGAGCGCCG (reverse). PCR reaction conditions were as follows: initial denaturation at 95°C for 2 min, 18 cycles of denaturation at 95°C for 15 sec, annealing at 55°C for 30 sec and elongation at 68°C for 5 min. All mutants were sequenced to validate the expected mutations (Robarts Research Center, London, Canada). Rac1 and all the Rac1 mutants were subcloned into pCMV-HA and EGFP-C3 using the ECoRI and BamHI restriction sites. CaM was cloned into mCherry-C1 and pCMV-HA vector using ECoR1 restriction sites and blunt ligation.

### Protein expression and purification

GST, GST-Rac1 wild type, GST-Rac1 mutants, GST-RhoA and GST-H-Ras were expressed in BL21 or DH5α *Escherichia coli* cells and purified using glutathione-agarose beads as described previously [19], [24]. The purity of the final preparations was assessed using SDS-PAGE and is included in the supplementary material (Figures S1, S2, S3).

### Interaction of Rac1 with pure CaM

Purified GST or GST-Rac1 or GST-Rac1 mutants bound to GSH-agarose beads were washed with MOPS buffer consisting of 30 mM MOPS (pH 7.2), 1% NP-40 and 100 mM KCl. In addition to buffer alone, 10 mM EGTA, 5 mM Ca^2+^ plus 20 µg of pure CaM were added to tubes containing GST or GST-Rac1 or GST-Rac1 mutants (100 µl) and incubated for 2 h at 4°C. After incubation, the beads were washed three times with MOPS buffer containing the appropriate concentration of EGTA and Ca^2+^. Laemmli's sample buffer was added to washed beads and heated at 100°C for 5 min. Western blotting was performed using anti-CaM antibody and visualized using ECL.

### Cell culture and transfection

HeLa cells were obtained from ATCC and maintained in DMEM (Invitrogen) supplemented with 10% FBS. Expression plasmids were transfected into HeLa cells using lipofectamine 2000 (Invitrogen). CHRF-288-11 cell line was obtained from ATCC and maintained in RPMI-1640 plus 10% FBS.

### CaM-Sepharose pull-down of HA-Rac1, HA-Rac1 mutants, GFP-Rac1, constitutively active GFP-Rac1 (G61V) and dominant negative GFP-Rac1 (T17N) from HeLa cell lysate

For pull-down assays HeLa cells expressing the appropriate Rac1 were washed twice in phosphate buffered saline (PBS) and lysed in 0.50 ml of CaM-binding buffer containing 20 mM HEPES (pH 7.4), 200 mM KCl, 1 mM MgCl_2_, 0.55% Triton X-100 and a protease inhibitor cocktail consisting of 2 μg/ml aprotinin, 5 μg/ml leupeptin, and 1 mM PMSF. The lysate was centrifuged at 14,000xg for 10 min at 4°C. After centrifugation, supernatant was incubated with 100 µl of CaM-Sepharose 4B beads. The reaction mixture was incubated for 2 h at 4°C and washed three times in CaM-binding buffer. Laemmli's sample buffer was added to beads and heated at 100°C for 5 min. Eluted proteins were subjected to 12% SDS-PAGE, electrophoretically transferred to PVDF membrane and western blotting performed using anti-HA or anti-GFP antibody.

### Inhibition of Rac1 activation by W7

To assess if CaM is required for Rac1 activation, HeLa cells or CHRF-288-11 cells were serum starved for 12 h followed by incubation with W7 (150 μM) for 10 min. The HeLa and CHRF-288-11 cells were stimulated with EGF (100 ng/ml) or thrombin (0.2 U/ml) respectively for the indicated times. The cells were lysed using RIPA buffer (50 mM Tris-HCl, pH 7.4, 1% Triton X-100, 0.5% sodium deoxycholate, 0.1% SDS, 500 mM NaCl, 10 mM MgCl_2_, 2.5 mM EGTA, and a protease inhibitor cocktail). The lysate was centrifuged at 14,000xg for 10 min at 4°C. After centrifugation, the supernatant was incubated with GST-PAK1 for 2 h at 4°C. After incubation, the beads were washed three times with Rac1 washing buffer (50 mM Tris-HCl, pH 7.4, 10 mM MgCl_2_, 150 mM NaCl, 1% Triton X-100, 5 mM EGTA and a protease inhibitor cocktail). The final bead pellet was suspended in 30 μl of Laemmli's sample buffer and heated at 100°C for 5 min. Western blotting was performed using mouse anti-Rac1 antibody.

### Activation by EGF of HA-Rac1 and HA-Rac1 mutants in HeLa cells

To assess Rac1 activation, plasmids for wild type HA-Rac1 or mutants were transfected into HeLa cells. 48 h post-transfection, cells were serum starved for 12 h and incubated in the absence or presence of EGF (100 ng/ml) for indicated times. The cells were lysed using buffer containing 50 mM Tris-HCl, pH 7.4, 10 mM MgCl_2_, 150 mM NaCl, 1% Triton X-100, 5 mM EGTA and a protease inhibitor cocktail. The lysate was centrifuged at 14,000xg for 10 min at 4°C. After centrifugation, the supernatant was incubated with GST-PAK1 for 2 h at 4°C and the beads were washed three times with washing buffer (25 mM Tris-HCl, pH 7.5, 1 mM DTT, 30 mM MgCl_2_, 40 mM NaCl, 0.5% NP-40, and a protease inhibitor cocktail). The final bead pellet was suspended in 30 µl of Laemmli's sample buffer and heated at 100°C for 5 min. Western blotting was performed using mouse anti-HA antibody.

To assess if higher amount of CaM is associated with Rac1 during EGF mediated activation of Rac1, HeLa cells overexpressing wild type HA-Rac1 were stimulated using EGF for varying times. Equal amount of lysate protein (500 µg) from various samples was incubated at 4°C for 2 h with anti-HA antibody coupled to agarose beads. The beads were washed, and SDS-PAGE and western blotting analysis was carried out using anti-CaM antibody.

### GTP loading of HA-Rac1 mutants

To confirm that HA-Rac1 mutants were able to bind GTP, loading of HA-Rac1 mutants expressed in HeLa cells with GTPγS or GDPβS was performed *in vitro*. HeLa cells expressing wild type HA-Rac1 or mutants were washed twice with PBS and lysed in buffer containing 50 mM Tris-HCl, pH 7.5, 2.5 mM MgCl_2_, 500 mM NaCl, 1% NP-40, 10 mM NaF, 10% glycerol, 1 mM sodium orthovanadate and a protease inhibitor cocktail. The lysate were centrifuged at 14,000xg for 10 min at 4°C. After centrifugation, 10 mM EGTA (final concentration) and guanine nucleotide (100 µM GTPγS or 100 µM GDPβS) were added to the supernatant and the mixture was incubated at 30°C for 15 min. At the end of the incubation, MgCl_2_ was added to achieve a final concentration of 60 mM to lock in nucleotides. The mixture was incubated with 100 µl GST-PAK1 for 2 h at 4°C. Unbound proteins were removed by washing three times with binding buffer. 30 µl of Laemmli's sample buffer was added to beads and heated at 100°C for 5 min. SDS-PAGE and western blotting was performed using anti-HA antibody.

### Confocal Imaging of Rac1 in HeLa cells

For visualizing in whole cells, HeLa cells were plated at 0.12×10^6^ cells per well in 8 chamber cover glass system (Nalge Nunc International) and co-transfected using Lipofectamine 2000 with mCherry-CaM and EGFP-Rac1 or mCherry-CaM and EGFP-Rac1 mutant (K153A or R163A or K153A/R163A). 48 h post transfection, the localization of Rac1, Rac1 mutants and CaM was detected using a confocal microscope system (Olympus FV500).

### Effect of CaM over-expression on endogenous Rac1 activity

To further assess the role of CaM in Rac1 activation, HeLa cells were transfected with HA-CaM plasmid, serum starved for 12 h and stimulated using EGF. Active Rac1 was pulled using GST-PAK1 and western blotting performed using anti-Rac1 antibody.

### Effect of CaM knockdown on Rac1 activation

CaM knockdown was achieved using short hairpin RNA (shRNA) technology. The target sequences for CaM2 and CaM3 were selected as described by Lee et al [25]. The target sequences are 5′-GCAGAGTTACAGGACATGATT-3′ (CaM2) and 5′-GCCAGGTCAATTATGAAGAGT-3′ (CaM3). HeLa cells were co-transfected with puromycin resistant CaM2 and CaM3 shRNA plasmids (Open Biosystems). We used non-target shRNA as the negative control. After 48 h, the cells were serum starved for 12 h and stimulated using EGF and active Rac1 pulled out using GST-PAK1. SDS-PAGE and western blotting was performed using anti-Rac1 and CaM antibodies.

### Interaction of Rac1 and CaM using Docking

To further confirm the interaction between Rac1 and CaM, we used ZDock server to analyze binding between CaM and Rac1. The Apo-CaM (PDB id: 1CFD) was docked with Rac1 151–164 amino acids of Rac1 WT (PDB id: 1FOE), Rac1 K153A, Rac1 R163A, and K153A and R163A using Z DOCK server (http://zdock.bu.edu/). The docking calculations were carried out using Fast fourier transform based protein docking method using ZDock. This involves searches of all possible binding modes in the translational and rotational space between two proteins and evaluates each by an energy scoring function. The poses with the best energy scores were chosen for further analysis. The models were visualized using PyMol (http://www.pymol.org/).

### Statistical analysis

Where required the autoradiograph was scanned and bands quantified using Bio-Rad “Quantity One” program. The output was normalized and descriptive statistical analysis and t-tests were performed.

## Results

### Rac1 and Rac1 mutants bind pure CaM

**Figure 1:**
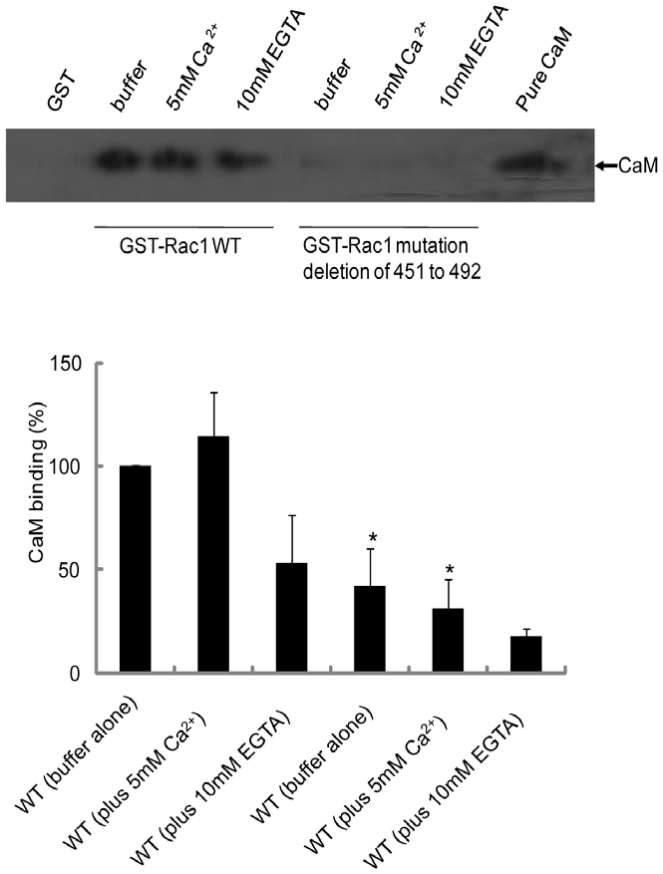
Effect of deletion of Rac1 putative CaM binding domain on interaction with purified bovine brain CaM. Equal amount (20 µg) of wild type (WT) GST-Rac1 and GST-Rac1 mutant (amino acids 151 to 164 deleted) were incubated with purified CaM (20 µg) in MOPS buffer and allowed to shake for 2 h at 4°C. GST beads were used as negative control. The incubation conditions were WT GST-Rac1 or GST-Rac1 mutant beads with buffer alone, buffer plus 5 mM Ca^2+^ or buffer plus 10 mM EGTA. At the end of the incubation, beads were washed three times and bound proteins were eluted using Laemmli's sample buffer. Western blot analysis was carried out using anti-CaM antibodies. A representative autoradiograph and quantitation is shown above. The experiment was repeated a minimum of three times.

We have previously shown that amino acid residues in the 151–164 region of Rac1 have the potential to bind CaM [24]. To further confirm this, we first tested whether removal of this region (amino acids 151–164) from Rac1 would affect CaM binding. Wild type and the GST-Rac1 mutant lacking region 151–164 were expressed in bacteria, purified, and incubated with pure bovine brain CaM. As shown in Figure 1, pure CaM bound to wild type Rac1 and the binding was enhanced by the addition of Ca^2+^ and reduced but not abolished by the addition of EGTA. In the mutant form lacking the CaM-binding region the interaction with CaM and Rac1 was reduced significantly (Figure 1). There was no binding of CaM to GST (Figure 1). These results confirm that amino acids in the 151–164 region of Rac1 play an important role in CaM interaction and that there may exist an additional CaM-binding site in Rac1 that is located in another region of Rac1. No interaction between CaM and RhoA or H-Ras (Figure S3) was observed confirming specificity of CaM interaction with Rac1 (Figure 1).

### Identification of critical residues in the CaM-binding domain of Rac1

**Figure 2:**
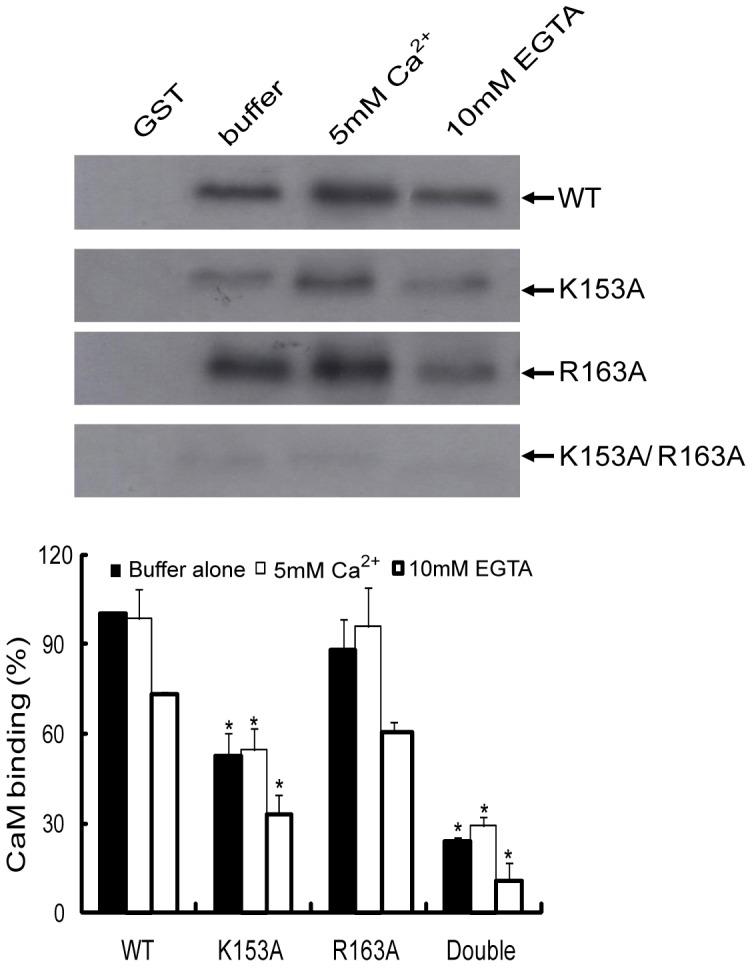
Binding of pure CaM to Rac1(K153A), Rac1(R163A), and Rac1(K153A/R163A). Equal amount (20 µg) of wild type (WT) GST-Rac1 and different GST-Rac1 mutants were incubated with purified CaM (20 µg) in MOPS buffer and allowed to shake for 2 h at 4°C. GST beads were used as negative control. The incubation conditions were beads containing WT GST-Rac1 or different GST-Rac1 mutants with buffer alone, buffer plus 5 mM Ca^2+^ or buffer plus 10 mM EGTA. At the end of the incubation, beads were washed three times and bound proteins were eluted using Laemmli's sample buffer. Western blot analysis was carried out using anti-CaM antibodies. Quantification was carried out using Bio-Rad Quantity one program and *p<0.05 values were considered significantly different (n = 3). In the figure above double refers to Rac1(K153A/R163A).

To identify critical residues in Rac1 that participate in the interaction with CaM, we used site-directed mutagenesis to change two positively charged amino acids (K153A and R163A) as basic amino acids are believed to be important in interaction of CaM with other proteins [14]. Thus, the wild type GST-Rac1, GST-Rac1 (K153A), GST-Rac1 (R163A) and GST-Rac1 (K153A and R163A) fusion proteins were expressed in *E.coli* and purified (Figures S1 and S2). Equal amounts of wild type GST-Rac1, GST-Rac1 (K153A), GST-Rac1 (R163A) and GST-Rac1 (K153A/R163A) fusion proteins coupled to GSH-agarose beads were used to pull-down pure bovine brain CaM *in vitro*. As shown in Figure 2, in the R163A mutant CaM binding was not affected, but in the K153A mutant CaM binding was significantly reduced. The binding of CaM was completely abolished in the double mutant K153A/R163A (Figure 2). These results demonstrate that amino acid K153 in Rac1 is essential for interaction with CaM. No binding of CaM was observed with GST beads alone.

### CaM-Sepharose pull-down of Rac1 from HeLa cells

**Figure 3:**
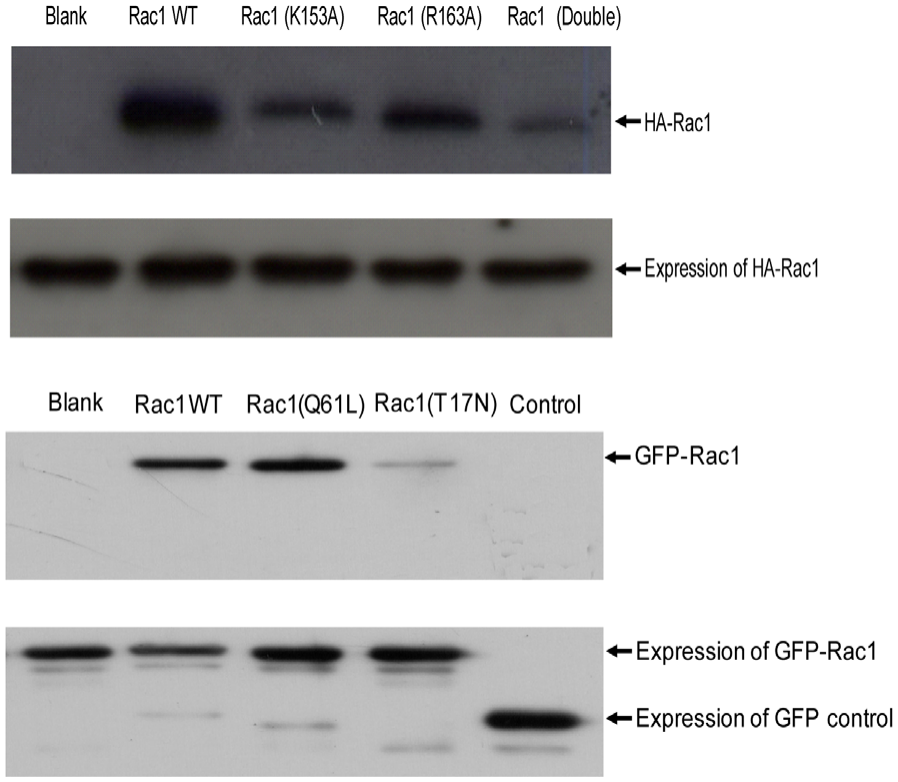
Rac1 mutants interact with CaM-Sepharose. (A) HA-Rac1 WT and HA-Rac1 mutants or (B) GFP-Rac1 wild type, GFP-Rac1(Q61L) and GFP-Rac1(T17N) were expressed in HeLa cells. CaM-Sepharose 4B beads (80 µl) were incubated with HeLa cell lysate (200 μg) expressing various forms of HA-Rac1 or GFP-Rac1 for 2 h at 4°C. At the end of incubation, CaM-Sepharose 4B beads were washed three times with binding buffer and bound proteins eluted using Laemmli's sample buffer. Western blot analysis was carried out using anti-HA antibody or anti-GFP antibody. Equal amount of HA-Rac1 or GFP-Rac1 proteins in lysates was confirmed using anti-HA antibody or anti-GFP antibodies. Quantification was carried out using Bio-Rad “quantity one ” program and *p<0.05 were considered significantly different (n = 3). In part (A) of the figure above double refers to the Rac1(K153A/R163A) mutant. The transient expression of the various versions of Rac1 proteins (lower panel, part A) and the GFP-Rac1 proteins (lower panel, part B) is also shown in the figure above.

To test whether the region of Rac1 which is important in binding to pure CaM *in vitro* is essential for interaction with CaM from cells, wild type HA-Rac1, HA-Rac1 (K153A), HA-Rac1 (R163A), and HA-Rac1 (K153A/R163A) fusion proteins were transiently expressed in HeLa cells and pull-down assays performed using CaM-Sepharose beads. As shown in Figure 3A, both the wild type HA-Rac1 and HA-Rac1 (R163A) interacted with CaM. However, HA-Rac1 (K153A) and the double mutant HA-Rac1 (K153A/R163A) demonstrated significantly reduced binding to CaM. These results further confirm the importance of amino acids K153 in Rac1 interaction with CaM and support the *in vitro* data obtained with interaction of Rac1 with pure CaM. The transient expression of the various forms of Rac1 proteins in HeLa cells is also shown (Figure 3A).

When CaM-Sepharose was used to pull-down constitutively active Rac1 (Q61L) or dominant negative Rac1 (T17N) expressed in HeLa cells, Rac1 and CaM interaction was stronger for the constitutively active Rac1 (Q61L) when compared to that with Wild-type Rac1 (Figure 3B). However, the dominant negative Rac1 (T17N) demonstrated significantly reduced interaction with CaM (Figure 3B). This result suggests that T17 in the N-terminus of Rac1 may also be important for interaction of Rac1 with CaM and that this interaction is potentially GTP-dependent and/or that T17 is important in interaction with CaM. The transient expression of various Rac1 proteins in HeLa cells is also shown (Figure 3B).

### Regulation of Rac1 activation by CaM

**Figure 4:**
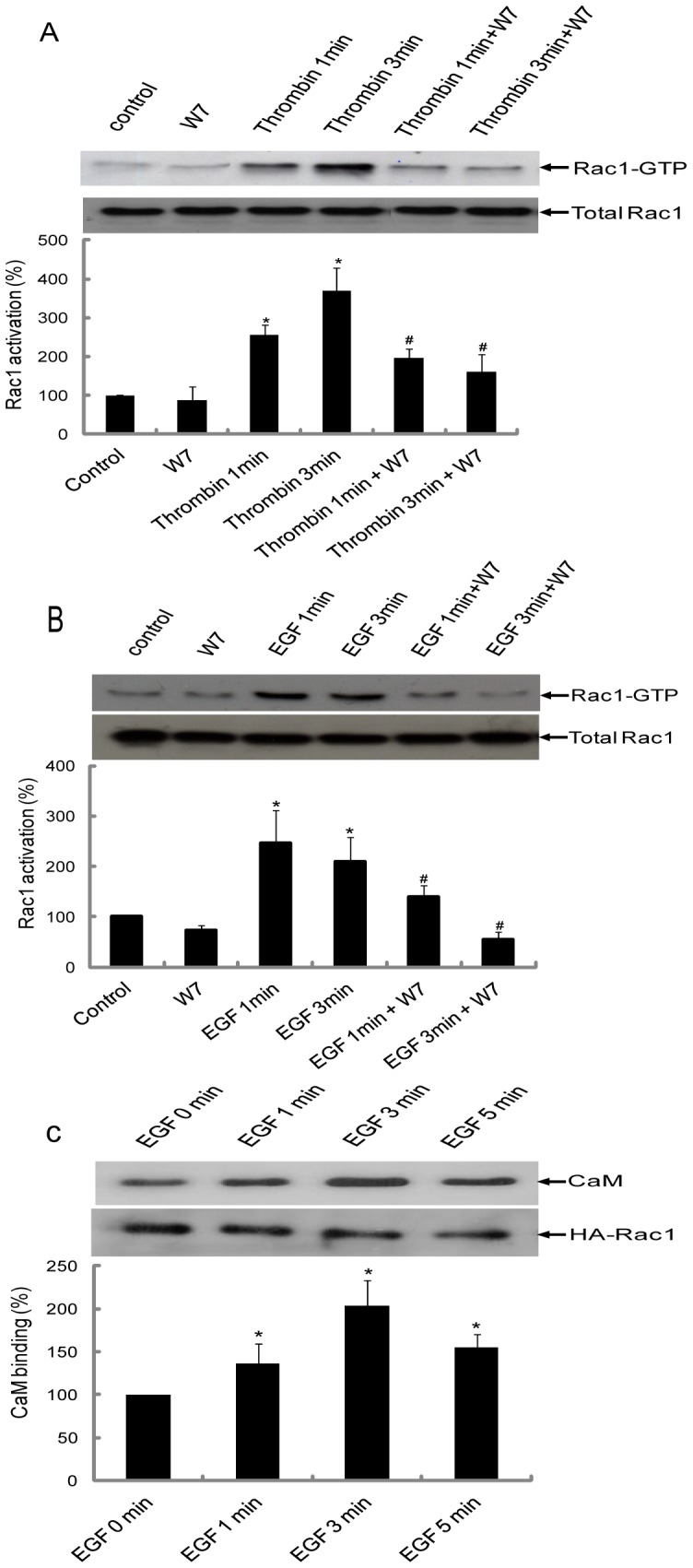
CaM is required for thrombin-induced activation of Rac1 in CHRF-288-11 cells and EGF-induced activation of Rac1 in HeLa cells. (A) CHRF-288-11 cells or (B) HeLa cells were serum starved for 12 h and incubated with W7 (150 μM) for 10 min followed by addition of thrombin to CHRF-288-11 cells and EGF to HeLa cells for 1 min or 3 min. At the end of the incubation cells were lysed using RIPA buffer. After centrifugation, 60 µl of the supernatant was suspended in 20 µl 4X Laemmli's sample buffer to determine level of endogenous Rac1 in various samples by western blotting. The rest of the supernatant was incubated with GST-PAK1 for 2 h at 4°C. After incubation, the beads were washed three times with Rac1 washing buffer. The final bead pellet was suspended in 30 µl of Laemmli's sample buffer and heated at 100°C for 5 min. Western blotting was performed using mouse anti-Rac1 antibody. Quantification (adjusted for endogenous level of Rac1) was carried out using Bio-Rad “quantity one” program and *p<0.05 were considered significantly different. #p<0.05 was considered significantly different compared with corresponding thrombin or EGF treatment. The experiments were repeated a minimum of three times. In part (C), an equal amount of lysate (500 µg) from HeLa cells transiently expressing wild type HA-Rac1 and stimulated for various times with EGF was incubated for 2 hrs at 4°C with anti-HA antibody coupled to agarose beads. At the end of incubation beads were washed and bound proteins analyzed using SDS-PAGE and western blotting using anti-CaM antibody. Quantification was carried out using Bio-Rad “quantity one” program and *p<0.05 were considered significantly different when compared to EGF at 0 min.

To examine the role of CaM in the regulation of Rac1 signaling, we treated CHRF-288-11 or HeLa cells with the CaM antagonist, W-7. Subsequently the cells were treated with thrombin (CHRF-288-11) or EGF (HeLa) for 1 min or 3 min. The Rac binding domain of the p-21 activated kinase (GST-PAK1) was used to assess the level of Rac1-GTP in cells. As shown in Figure 4A, thrombin significantly enhanced activation of Rac1 at 1 min and 3 min in CHRF-288-11 cells. This thrombin-induced Rac1 activation was abolished in the presence of the CaM inhibitor, W7. In a similar fashion, EGF caused activation of Rac1 in HeLa cells and this activation was also blocked in the presence of W7 (Figure 4B). Further, immunoprecipitation of Rac1-CaM complex was carried out at various time points after EGF stimulation. The results demonstrated that higher amounts of CaM is associated with Rac1 at the later time points after stimulation with EGF (Figure 4C).

### Role of CaM binding in Rac1 activation

**Figure 5:**
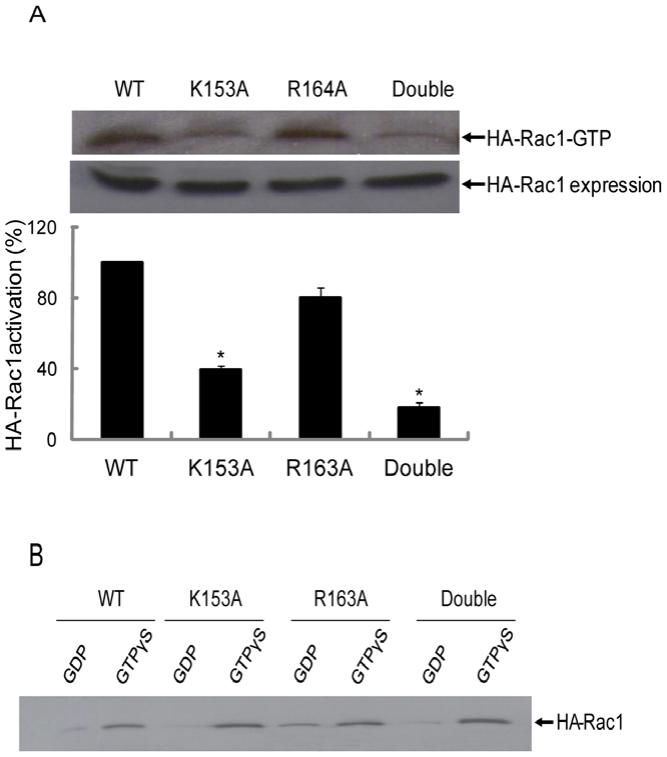
Activation of HA-Rac1 mutants is induced by EGF in HeLa cells. (A) Various HA-Rac1 mutants were transiently expressed in HeLa cells. 48 h post transfection cells were serum starved for 12 h and stimulated for 3 min with EGF and lysed as described in Materials & Methods. After centrifugation, the supernatant was incubated with GST-PAK1 for 2 h at 4°C and the beads were washed three times with washing buffer. The final bead pellet was suspended in 30 µl of Laemmli's sample buffer and heated at 100°C for 5 min. Western blotting was performed using mouse anti-HA antibody. Data presented is a representative immunoblot of at least three independent experiments. Quantification was carried out using Bio-Rad “quantity one” program and key * p<0.05 were considered significantly different. (B) GTP loading of WT Rac1 and mutants of Rac1 was tested by the GST-PAK1 pull-down assay. HeLa cells expressing various forms of HA-Rac1 were lysed in buffer as described in Materials & Methods. After centrifugation, guanine nucleotides (100 µM GTPγS or 100 µM GDPβS) plus 10 mM EGTA (final concn.) were added to the supernatant and the mixture was incubated at 30°C for 15 min. At the end of the incubation, magnesium chloride (MgCl_2_) was added to a final concentration of 60 mM to lock in nucleotides. The mixture was incubated with 100 µl GST-PAK1 beads for 2 h at 4°C. Unbound proteins were removed by washing three times with binding buffer. 30 µl of Laemmli's sample buffer was added to beads and heated at 100°C for 5 min. Western blotting was performed using mouse anti-HA antibody. In the figure above double refers to the Rac1 (K153A/R163A) mutant.

Since activation of Rac1 is regulated by CaM in HeLa cells we next investigated activation of the mutant forms of Rac1 where CaM binding was diminished. HeLa cells were transfected with wild type HA-Rac1 and mutant forms of HA-Rac1 to assess their activation in response to EGF. The results showed that wild type Rac1 and mutant Rac1(R163A) were activated upon treatment of cells with EGF (Figure 5A). However, the activation of mutant Rac1(K153A) and the double mutant Rac1(K153A/R163A) in response to EGF was significantly decreased compared to the wild type Rac1 and mutant Rac1(R163A) (Figure 5A).

The possibility existed that the mutant forms of Rac1 were unable to bind GTP and this was the reason that they were not activated. Thus, we tested the GTP loading capacity of the various Rac1 forms. HeLa cell lysates expressing wild type HA-Rac1 and HA-Rac1 mutants were incubated with GTPγS or GDPβS prior to incubation with GST-PAK1. The results demonstrated that GST-PAK1 pulled out HA-Rac1 that was loaded with GTPγS and not with GDPβS (Figure 5B). All forms of the Rac1 could be loaded with GTP (Figure 5B) suggesting that lack of activation was not due to the inability to exchange GTP for GDP.

### CaM and Rac1 localization in HeLa cells

**Figure 6:**
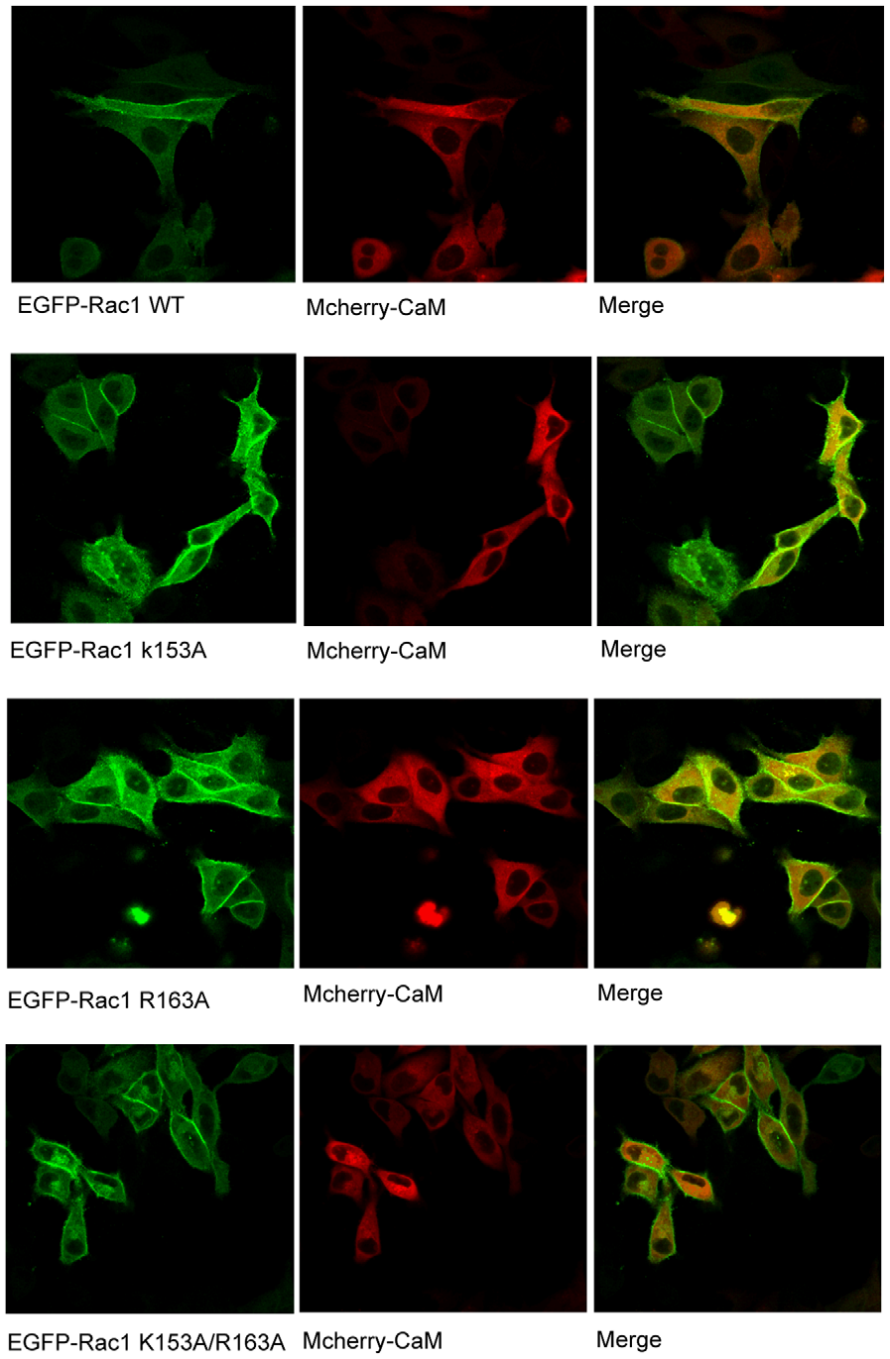
Localization of Rac1 and CaM in HeLa cells. HeLa cells were cotransfected with EGFP-Rac1 and mCherry-CaM or EGFP-Rac1 mutants and mCherry-CaM with Lipofectimine 2000. 48 h post-transfection, Rac1 and CaM localization was detected using a confocal microscope.

We have previously shown that CaM and Rac1 can be co-immunoprecipitated from platelets [24]. To demonstrate this interaction in live cells, we further analyzed co-localization of these proteins using confocal microscopy. HeLa cells were co-transfected with EGFP-Rac1WT and mCherry-CaM or EGFP-Rac1 mutants and mCherry-CaM and the localization of Rac1 and CaM in live cells was analyzed by confocal microscopy. In HeLa cells, EGFP-Rac1WT was primarily expressed around the plasma membrane with minor amounts in the cytoplasm (Figure 6A). mCherry-CaM was ubiquitously distributed in HeLa cells (Fig. 6A). Co-localization of EGFP-Rac1WT and mCherry-CaM was observed mostly around the plasma membrane (Figure 6A). The distribution of EGFP-Rac1 mutant (K153A), EGFP-Rac1 mutant (R163A) and EGFP-Rac1 mutant (K153A/R163A) was similar to that of EGFP-Rac1WT (Figures 6B, 6C and 6D). This indicated that the distribution of Rac1 was not affected due to amino acid changes.

### Effect of CaM overexpression and knockdown on Rac1 activation

**Figure 7:**
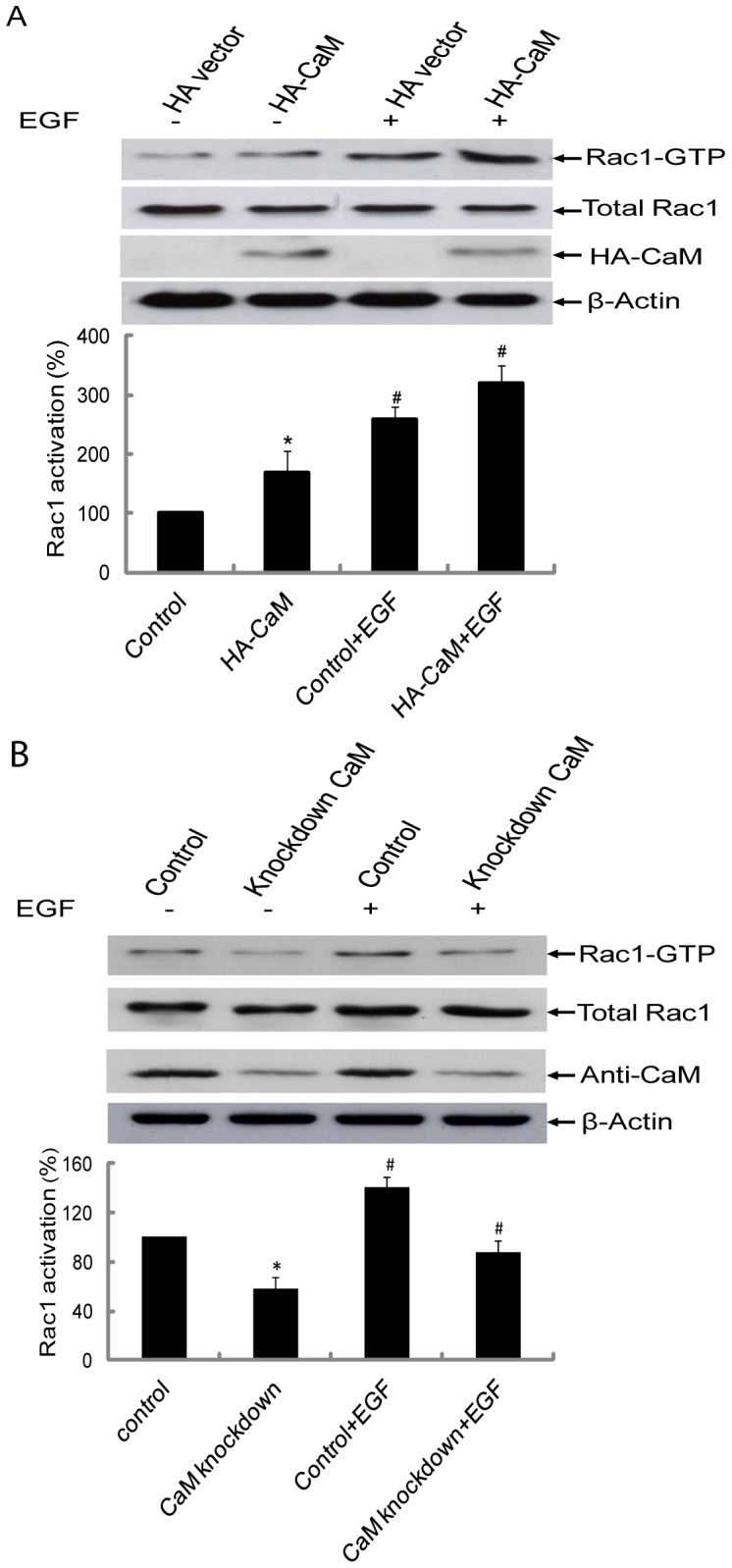
Effect of CaM over-expression or down-regulation on activation of Rac1. (A) Control HeLa cells and HeLa cells transiently over-expressing HA-CaM were serum starved for 12 h and stimulated with EGF for 3 min and analyzed for level of Rac1-GTP using GST-PAK1 pull-down assay. The total lysates were subjected to SDS-PAGE and immunoblotting performed using anti-Rac1 antibody. Anti-HA antibody was used to detect HA-CaM expression. β-actin antibody was used to establish equal loading of protein in different samples. Data presented is a representative immunoblot of at least three independent experiments. Quantification was carried out using Bio-Rad “quantity one” program and *p<0.05 were considered significantly different. #p<0.05 was considered significantly different compared with corresponding non-treatment samples. (B) HeLa cells were stably co-transfected with shRNAs targeting human CaM2 and human CaM3 or non-target shRNA as a negative control. HeLa cells were serum starved for 12 h and stimulated with EGF for 3 min. The level of Rac1-GTP was assessed using GST-PAK1 pull down assay. The total lysates were subjected to SDS-PAGE and immunoblotting using anti-Rac1 antibody or anti-CaM antibody or β-actin antibody. Data presented is one representative immunoblot of at least three independent experiments. Quantification was carried out using Bio-Rad “quantity one” program and *p<0.05 were considered significantly different. #p<0.05 was considered significantly different compared with corresponding non-treatment sample.

To further confirm a role for CaM in Rac1 activation, we analyzed the effect of CaM over-expression and knockdown on the activation status of Rac1 using GST-PAK1. HeLa cells over-expressing CaM as indicated by the presence of HA-CaM demonstrated a higher basal level of the active form of Rac1 when compared to control and this was further enhanced by treatment of cells with EGF (Figure 7A). Furthermore, knockdown of CaM using shRNA approach decreased level of GTP-bound Rac1 activity in un-stimulated and EGF stimulated cells when compared to control (Figure 7B).

### Analysis of Rac1 and CaM interaction using ZDock

**Figure 8:**
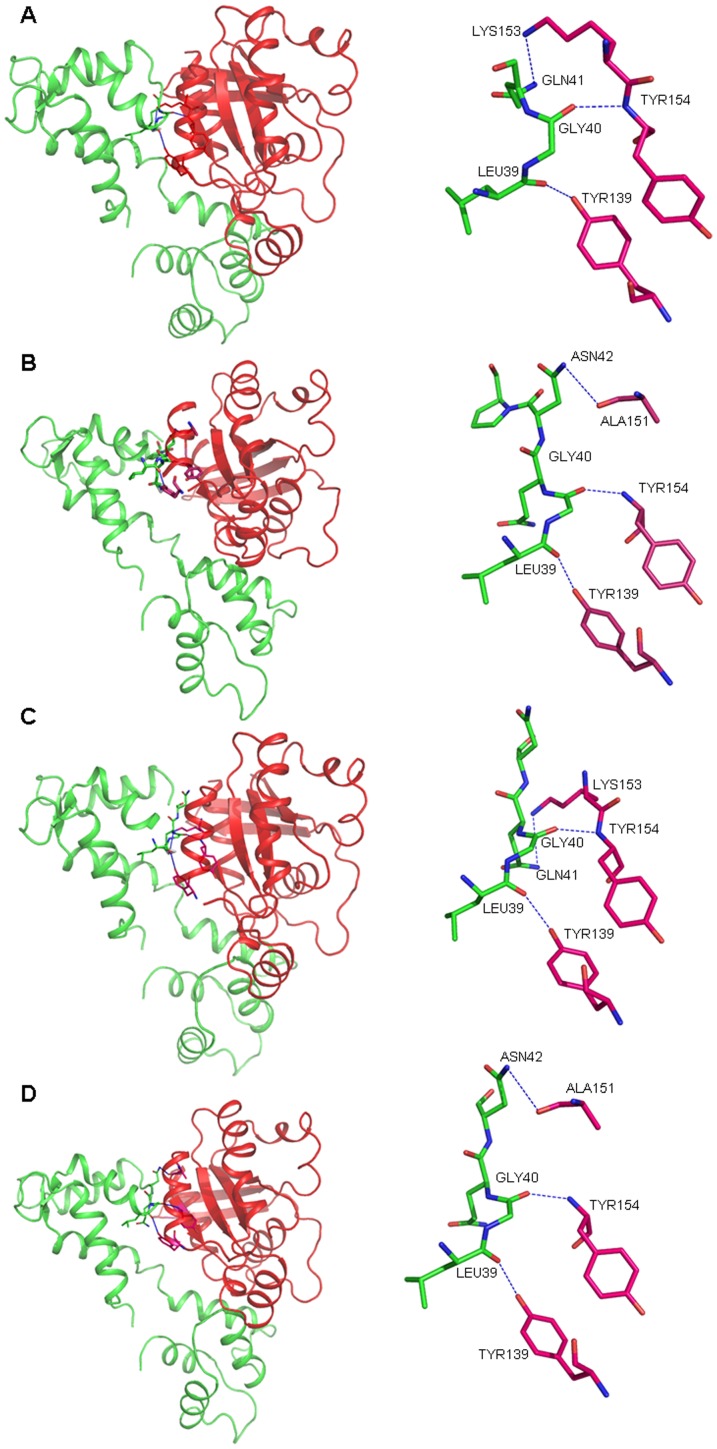
Predicted model for the interaction between various forms of Rac1 and CaM. The Apo-CaM (PDB id: 1CFD) was docked with Rac1 151–164 amino acids of Rac1 WT (PDB id: 1FOE), Rac1 K153A, Rac1 R163A, and K153A/R163A using ZDock server (http://zdock.bu.edu/). The docking calculations were carried out using Fast fourier transform based protein docking method using ZDock. All possible binding modes in the translational and rotational space between two proteins were searched and each was evaluated by an energy scoring function. The poses with the best energy scores were chosen for further analysis. The models were visualized using PyMol (http://www.pymol.org/). CaM is shown in green and Rac1 is in red. The figure above represents interaction between: (A) WT Rac1 and CaM; (B) Rac1 K153A and CaM; (C) Rac1 R163A and CaM and (D) Rac1 K153A/R163A and CaM.

To further assess interaction between Rac1 and CaM, we used ZDock server to analyze binding between CaM and Rac1. The results showed that Rac1 K153 directly interact with CaMQ41 (Figure 8A). After K153 was mutated to alanine in Rac1, the interaction between K153A and CaMQ41 was abolished (Figure 8B). However, a new interaction between Rac1A151 and CaMN42 was observed (Figure 8B). In the R163A mutant there was no change in the interaction between K153 and Q41 (Figure 8C). Double mutant K153A and R163A demonstrated the same interaction pattern as the K153A mutant (Figure 8D).

## Discussion

CaM is a major calcium sensor protein that plays an important role in Ca^2+^-dependent signaling pathways by regulating the activity of structurally and functionally distinct target proteins [13]. Of particular interest are the recent observations that CaM interacts with a variety of small GTPases [19]–[24]. CaM was shown to be necessary for the thrombin-induced activation of RalA and RalB in human platelets [19] and that W7, a CaM inhibitor, blocked activation of the small GTPase Rac1 in neutrophils [26]. We further investigated the role of CaM and demonstrated that CaM was able to directly bind Rac1 and that this was important in the activation step of Rac1 in human platelets and CHRF-288-11 cells [24], [27]. In addition, we showed that amino acids in the region 151–164 in Rac1 were important in binding CaM [24]. However, the role of individual amino acids in this region is not known both in terms of binding to CaM and in Rac1 activation. In this study, we have attempted to address these questions.

The sequence homology among the CaM targeted sequences is low with the binding domain of many CaM regulated proteins containing a short peptide of 16–35 residues, in which amphipathic α-helices are formed with hydrophobic and basic residues being critical for binding of CaM-binding proteins to CaM [14], [28]. The potential CaM-binding region encompassing amino acids 151-164 in Rac1 contained 57% hydrophobic residues and basic amino acids at position 153(K) and 163(R). Deletion of the region 151–164 in Rac1 significantly reduced binding to pure CaM (Figure 1). However, this deletion did not result in total loss of CaM binding to Rac1 suggesting that Rac1 may potentially have two binding sites for CaM. A similar situation has been reported previously for the Ral GTPase where we demonstrated that RalA and RalB have an N-terminal Ca^2+^-independent and a C-terminal Ca^2+^-dependent binding site for CaM [19]. The results from dominant negative Rac1 (T17N) support this suggestion as this form of Rac1 does not bind CaM (Figure 3B). The lack of binding to Rac1T17N could also mean that CaM interacts with the GTP-bound form of Rac1.

To further investigate the role of individual amino acids in CaM binding and Rac1 activation, the basic amino acid 153K and 163R were mutated to neutral amino acid, alanine. The Rac1 mutant K153A demonstrated reduced binding to CaM while the Rac1 mutant R163A did not demonstrate any change in CaM binding when compared to wild type Rac1 (Figure 2). Similar results were obtained when the Rac1 mutants were expressed in cells and pulled out from the lysate using CaM-Sepharose (Figure 3A). The double Rac1 mutant (K153A/R163A) demonstrated essentially no binding to CaM (Figures 2 and 3A) suggesting some cooperation between these two residues. To further confirm interaction between Rac1 and CaM, we used ZDock server to analyze interaction between CaM and Rac1. The result showed that Rac1 K153 directly interacts with Q41 in CaM (Figure 8A). Hydrogen bonding region in CaM occurs between the N-terminal helix I and the final EF hand helices VII and VIII in the c-domain as well as the helices of the collapsed linker [29]. Other resides at the ends of the loop in each domain (Gln41 and Gln 114) are also involved in hydrogen bond formation [29]. These data points to the importance of amino acid K153 in Rac1 in binding to CaM and supports the role of basic amino acids in interaction of CaM with its target protein. However, just the mere presence of a basic amino acid in the CaM-binding region does not suggest its role in CaM binding since basic amino acid R163 in Rac1 does not play a major role in CaM binding. Other factors could also be of importance in CaM binding. We had previously demonstrated that CaM binding to small GTPase Ral required the isoprenlylated form of Ral [30]. More recently, it was demonstrated that farnesylation of K-Ras and the polybasic region are both essential for interaction between K-Ras and CaM [28]. Vidal-Quadras [31] demonstrated that hypervariable region of Rac1 specially the polybasic region and the adjacent prenyl hydrophobic group in the GTP-bound state of Rac1 are essential for CaM binding in a Ca^2+^ dependent manner. Different domains of Rac1 may co-operate in CaM binding and that the polybasic region and prenyl group are both essential but not sufficient to accomplish CaM interaction [31].

Previously we used human platelets as the model system to demonstrate that CaM inhibitor W7 reduces Rac1 activation [24]. In this study, the role of CaM in Rac1 function was further examined using HeLa cells and the megakaryocytic cell line, CHRF 288-11. The optimal time point for Rac1 activation in CHRF-288-11 cells by thrombin (Figure 4A) was similar to that observed in platelets [24] and this activation was inhibited by W7 (Figure 4A). The time course of Rac1 activation in HeLa cells peaked at 1min and decreased by 3 min (Figure 4B). This is similar to that reported by other investigator for Rac1 activation in Cos1, A431, and NIH3T3 cells in response to EGF [32]–[34]. The activation of Rac1 in HeLa cells was also inhibited by W7 (Figure 4B). The role of CaM in Rac1 activation was further examined using Rac1 mutants. EGF stimulation of wild type Rac1 or Rac1(R163A) in HeLa cells led to the activation of Rac1 (Figure 5A). In contrast, Rac1 mutants K153A and double mutant K153A/R163A demonstrated significantly reduced Rac1 activation in response to EGF (Figure 5A). These results mirror the ability of mutants to bind CaM (Figure 2). The Rac1 mutants that did not bind CaM (K153A and K153A/R163A) also demonstrated reduced Rac1 activation. This lack of activation was not due to an inability to exchange GTP for GDP (Figure 5B) or due to mislocalization of Rac1 as the different Rac1 mutants used in the current study demonstrated subcellular localization that was similar to that of the wild type Rac1 (Figure 6). Thus, CaM does not play a role in the subcellular localization of Rac1 even though a role for CaM in the localization of K-Ras has been proposed [35]. The mechanism of by which CaM regulates Rac1 activity remains to be elucidated. It has been shown that EGF signal leading to the activation of Rac1 was transduced from EGFR by GEFs such as Asef, Sos, Tiam1, and Vav2 [33], [36]–[38]. A GEF like role for CaM in the regulation of Ral GTPase activation has also been proposed [39]. We anticipate that CaM might act as a GEF in controlling Rac1 activation. A recent study showed that Tiam2, a GEF for Rac1, was down-regulated by CaM knockdown in cortical neurons [40]. This would suggest that results obtained using CaM knockdown (Fig. 7B) can be explained due to changes in the level of GEFs for Rac1. Another possible mechanism to explain regulation of Rac1 activation by CaM is through the inhibition of Rac1-GAP function by CaM. Consequently, CaM will inhibit Rac1-GAP-mediated inactivation of Rac1 and thus leaving the GTPase in the GTP-bound form. However, the precise mechanism by which CaM regulates Rac1 activity and the functional role of this interaction still needs to be resolved.

The present study has confirmed that CaM plays a critical role in the regulation of Rac1 activity and that amino acid K153 in Rac1 is important for CaM binding. In addition, amino acid K153 in Rac1 is also required for CaM-mediated activation of Rac1 in response to external stimuli. These results further demonstrate an obligatory role for CaM in the activation of Rac1 and as a consequence a role in cytoskeleton reorganization and cell migration. Further studies are required to define precisely the role of CaM in Rac1 function.

## Supporting Information

Figure S1 **GST, GST-Rac1 WT and GST-Rac1 mutant (amino acid 151–164 deleted) were expressed as fusion proteins in** ***Escherichia coli*** **and purified using GSH-agarose beads as described in “Materials and Methods”.** An aliquot of the beads was analyzed using SDS-PAGE. A representative picture of a Coomassie blue stained gel is shown above.
(DOCX)

Figure S2 **GST, GST-Rac1 WT, GST-Rac1 (K153A), GST-Rac1 (R163A) and GST-Rac1 (K153A/R163A) were expressed as fusion proteins in** ***Escherichia coli*** **and purified using GSH-agarose beads as described in “Materials and Methods”.** An aliquot of the beads was analyzed using SDS-PAGE. A representative picture of a Coomassie blue stained gel is shown above.
(DOCX)

Figure S3 (A) GST, GST-RhoA, GST-H-Ras and GST-Rac1 WT were expressed as fusion proteins in *Escherichia coli* and purified using GSH-agarose beads as described in “Materials and Methods”. An aliquot of the beads was analyzed using SDS-PAGE. A representative picture of a Coomassie blue stained gel is shown above. (B) An aliquot of agarose beads (50 µl) containing the various GST fusion proteins was incubated for 2 h at 4°C with pure CaM (20 µg) as described in “Materials and Methods”. At the end of the incubation the beads were washed and analyzed by SDS-PAGE and western blotting using anti-CaM antibody.
(DOCX)
